# Freeze-Damage Detection in Lemons Using Electrochemical Impedance Spectroscopy

**DOI:** 10.3390/s19184051

**Published:** 2019-09-19

**Authors:** Adrián Ochandio Fernández, Cristian Ariel Olguín Pinatti, Rafael Masot Peris, Nicolás Laguarda-Miró

**Affiliations:** 1 Escuela Técnica Superior de Ingeniería del Diseño, Universitat Politècnica de València, Camí de Vera s/n, 46022 Valencia, Spain; adocfer@etsid.upv.es; 2 Instituto Interuniversitario de Investigación de Reconocimiento Molecular y Desarrollo Tecnológico (IDM), Unidad Mixta Universitat Politècnica de València—Universitat de València, Camí de Vera s/n, 46022, Valencia, Spain; criolpi@upvnet.upv.es (C.A.O.P.); ramape@eln.upv.es (R.M.P.)

**Keywords:** electrochemical impedance spectroscopy, lemon, freeze damage, detection

## Abstract

Lemon is the most sensitive citrus fruit to cold. Therefore, it is of capital importance to detect and avoid temperatures that could damage the fruit both when it is still in the tree and in its subsequent commercialization. In order to rapidly identify frost damage in this fruit, a system based on the electrochemical impedance spectroscopy technique (EIS) was used. This system consists of a signal generator device associated with a personal computer (PC) to control the system and a double-needle stainless steel electrode. Tests with a set of fruits both natural and subsequently frozen-thawed allowed us to differentiate the behavior of the impedance value depending on whether the sample had been previously frozen or not by means of a single principal components analysis (PCA) and a partial least squares discriminant analysis (PLS-DA). Artificial neural networks (ANNs) were used to generate a prediction model able to identify the damaged fruits just 24 hours after the cold phenomenon occurred, with sufficient robustness and reliability (CCR = 100%).

## 1. Introduction

Lemon (*Citrus Limon* (L.) Osbeck) is one of the world’s most remarkable citrus crops, with a total production of almost 16 million tons in 2016, representing 12.2% of the total world citrus production. Spain, with 950 thousand tons, is the sixth country in terms of lemon production and the second one in terms of export, dedicating two thirds of its production to the international market and being second only to China [1]. Thus, the cultivation, postharvest and commercialization of lemons activities are of strategic importance in Spain.

However, lemon production and export present a problem related to freeze damage [2,3]. Periodically, the Mediterranean region (where most of the Spanish lemon is produced) experiences adverse climatic phenomenon resulting in chilling nights. Additionally, an inadequate management of refrigerated storage may also result in freezing. Both cases generate a loss in quality and potential commercialization of citrus fruits [4,5,6]. In general terms, freeze damage in lemons appears when temperatures fall below between −0.8 and −1.4 °C, as the lemon is the most sensitive citrus fruit to congelation [7,8]. However, the consequences of chilling may vary depending on the intensity and duration of the freezing episode and other bio-climatic factors [9].

In fact, freezing affects in many ways. If the phenomenon is rapid, ice crystals appear both in the interstices among the cells and inside them, thus, breaking the structure and causing cellular death, which causes necrosis in the affected tissue affected and the impossibility of any type of recovery. On the other hand, if freezing is slow enough, intercellular water begins to freeze such that the cells are not initially affected. If the phenomenon remains, a progressive dehydration of the cell will take place due to the loss of water towards the interstitial, leading to osmotic equilibrium between liquids in the inner and outer part of the cells. This process, if it is too demanding for the cell, can lead to death, but only if it exceeds its ability to yield water without perishing. Additionally, thawing after congelation can lead to the survival of living cells if it is slow enough. However, if it is too fast, it can also lead to dehydration and cell death by transpiration [9,10].

Additionally, some authors have identified specific effects of freezing on lemons such as textural loss and soaked appearance, the disruption of the normal metabolism of the fruit [11,12], the liberation of enzymes, changes in color, odor, flavor and nutritive value [13], pitting, rind staining, red blots and necrosis on the flavedo [14]. Some of these effects are easily detectable, but others are neither obvious nor immediate in appearance, so they are difficult to identify.

In order to solve this problem, several methods for freeze-damage detection and control in citrus fruits have been developed. These methods are diverse, ranging from the simplest—based on the visual inspection of the fruit [15]—to physical techniques such as the separation of the fruit by density (flotation) [16], ethanol detection [5], vision sensors [17,18], gas-mass chromatography [19], fluorescence [6] or nuclear magnetic resonance [20]. These methods are mostly laboratory techniques that have specific requirements in terms of instruments, personnel, time, sampling and testing, thus making them comparatively less agile with respect to electrochemical impedance spectroscopy (EIS).

On the contrary, EIS, combined with an adequate statistical treatment of the data, is a simple, inexpensive, immediate, on-line and robust technique [21,22]. Impedance spectroscopy [23] is a method for characterizing the electrical properties of materials and their interfaces using electrodes. This technique involves applying an electrical stimulus to electrodes, observing the response and determining their properties, interactions and dependencies with certain factors. The electrical response can vary substantially depending on the charges (free ions), microstructure, and nature of the sample and geometry and properties of the electrodes. As electrical impedance is an intrinsic property correlated with the internal structure of the samples, EIS measurements [24] determine or infer information about them as long as the event in analysis presents a change in their electrical behavior. In fact, EIS has already been successfully used in the field of food technology, particularly in the quality control of several parameters of fruits and vegetables [25,26] such as bananas [27], kiwis [28], mangoes [29], eggplants [24], tomatoes [30], carrots [31,32] potatoes [33], manufactured products [34] and their waste valorization [35,36].

Knowing the impedance value of the samples and the processes they have experienced, statistical prediction models can be obtained by means of multivariate analysis techniques that can later be used to predict the properties of new samples from their corresponding impedance measurements. Given that EIS generates a large amount of data per test, a powerful statistical tool is necessary to ensure reliable results. Consequently, principal components analysis (PCA) and partial least squares (PLS) have been used in a first phase, as they have thus far given very satisfactory results when working with this type of data [37,38,39]. Here, a discriminant analysis by partial least squares was selected, as there was a large amount of data per sample, and it was clearly organized in groups with different electrochemical responses [25,40]. Next, artificial neural networks (ANNs) were used to improve the aforementioned methods since they are very flexible and adaptable, easily fitting to non-linear systems and able to learn from their own mistakes [41,42]. ANNs are also easy to use, clear, and easily implementable on a personal computer (PC) since they also have low computational requirements. In fact, the potential application of ANNs that can be implemented in a microprocessor to create a portable detection device that could be used in the field for in-situ freeze damage detection was of particular interest for this study.

Accordingly, and attending to the preliminary experience in this field [25], the goal of this study was to determine the ability of a system combining EIS analysis by using a specific sensor and an adequate data treatment tool (ANN) to detect freeze-damage in lemons in a rapid, easy, economic and reliable way. 

## 2. Materials and Methods

### 2.1. Raw Material

Lemons were selected based on their physical aspects, variety, origin, size and ripening, and the absence of damage or injuries; we tried to get a series of samples as homogeneous as possible according to their natural origin [43]. Once in the laboratory, fruits were washed, dried and kept at room temperature to be later subjected to the corresponding tests.

In the specific case of assays with frozen lemons, fruits were first exposed to a laboratory freezing night simulation by introducing them in a freezer (LIEBHERR Model GGU 1500 Premium, Liebherr-International Deutschland GmbH, Biberach an der Riß, Germany) long enough to reach and slightly exceed the freezing temperature of the fruit. Then, after the temperature stabilization of the product for at least 12 hours, they were tested again following the same protocol [25]. 

### 2.2. Electrochemical Impedance Spectroscopy System

**Figure 1:**
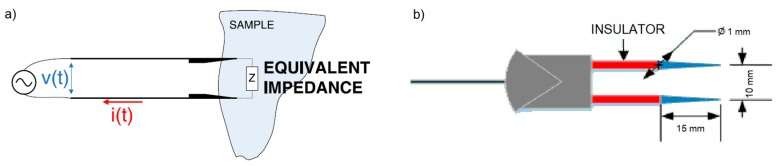
Schemes of (**a**) the applied two-electrode measurement technique and (**b**) the stainless-steel electrodes used in the assays.

The measurement technique used was two-electrode impedance spectroscopy (Figure 1a). This technique consists of applying a potential difference between two electrodes and measuring the current through them in order to find the electrode–sample–electrode impedance. The electrodes used are stainless steel needles. The material has a high corrosion resistance and is widely used in food contact applications [44]. In addition, the resistance of the electrodes is very small (~ 3 mΩ), in the order of about one million times smaller than the sample resistance, so the resistive part of measured impedance is practically the sample resistance. The electrodes were connected to two wires housed in an epoxy resin frame (Figure 1b).

**Figure 2:**
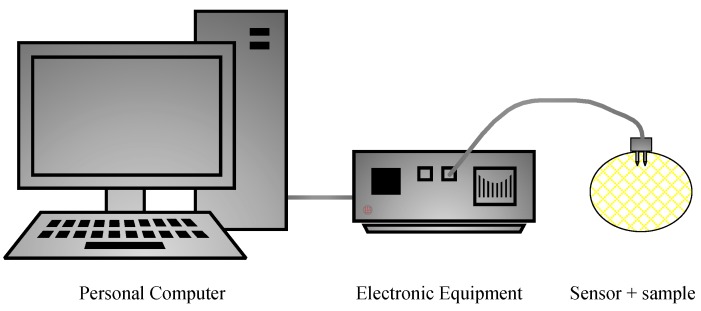
Electrochemical impedance spectroscopy system.

The system consisted of an electronic equipment and a software application that ran on a PC (Figure 2). Sinusoidal alternating electrical signals with different frequencies were applied to the sample, and the current response for each one of the frequencies was measured. Then, the system calculated the impedance spectrum of the sample by means of the Discrete Fourier Transform (DFT) and displayed it on the screen. The parameters of the system (signal amplitude, frequency range, current scale, etc.) were configured by the user through the graphical user interface. The EIS system was divided into two clearly differentiated parts.

#### 2.2.1. Software Application

A software application ran on a PC. It carried out a frequency sweep to obtain the impedance modulus and the phase of the sample. The user established the frequency range and the amplitude of the sinusoidal signals applied to the electrodes. For each one of the frequencies, the application calculated the signal temporal evolution and sent this information (along with the rest of the data needed to generate the signal) to the electronic equipment through a USB port. Then, with the data response of the electronic equipment, the software application determined the amplitude and the phase of the voltage and current signals through a DFT. From these data, the software application calculated the modulus and phase of the sample impedance for the current frequency. Then, the application stored the result of the measurement in a file and repeated the same process for the rest of the frequencies. The specifications of the EIS measurement system are shown in Table 1.

#### 2.2.2. Electronic Equipment

The electronic equipment received the information sent by the computer, generated the corresponding sinusoidal waveform and applied it to the sample. Then, the current and voltage signal responses were sampled and sent to the PC. For the receiving process of the data sent by the PC and the signal generation, the equipment used a complex programmable logic device (CPLD, Altera EPM7160SLC84), a 10-bit digital analogue converter (DAC) and a static 2 KB (2048 bytes) random access memory (RAM). A second CPLD, two 8-bit digital analogue converter and a configurable current sensor sampled the signals corresponding to the voltage applied to the electrode and the current flowing through it. The samples were stored in others two static RAM memories. Once a complete cycle of the signal was sampled, the values are transmitted to the PC.

The electronic measurement system was designed by the Group of Electronic Development and Printed Sensors (GED and PS) of the Interuniversity Institute for Molecular Recognition and Technological Development (IDM) at the Universitat Politècnica de València (UPV) [45].

### 2.3. Electrochemical Impedance Spectroscopy Analyses

Samples were previously prepared as described in Section 2.1. Then, ten fruits were selected, and EIS analyses were carried out. To do so, each lemon was tested in three different ways, conducting three repetitions and three iterations per test, taking a total of 27 assays per fruit. The first type of assays was conducted with unaltered lemons, puncturing the sensor directly into the skin. The second ones were made after peeling a part of the fruit and puncturing the sensor into a single segment. Finally, the third type of assays was performed by puncturing each needle (electrode) of the sensor into a different segment, leaving the segments membranes (separation between segments) approximately in the middle. The idea of conducting these three different kinds of tests was to determine which part of the fruit experienced the most significant changes in terms of electrochemical response to the frost phenomenon. In simple terms, this allowed us to know which part of the lemon was the most sensitive to freeze.

A specific protocol for the impedance spectroscopy tests was designed. It was as follows: Firstly, it was verified that the whole system (measurement device and PC) was turned on, connected and correctly working. Then, temperature of the fruit was measured by using a multimeter (FLUKE 16 Multimeter, FLUKE, Everett, WA, USA). Next, the sensor was punctured into the fruit in the appropriate way for the type of test to be conducted. Afterwards, the test prepared by activating the software. Then, the system received the order to generate a specific electric signal that was transmitted to the fruit via the sensor. The response was also collected from the sensor and arrived to the PC where it was shown on the screen and stored for the further processing of data. The sensor was then cleaned and dried to make it ready for the next test.

### 2.4. Data Treatment

Given that the response for each EIS test carried out consisted of a total of 100 data (50 modulus values and 50 phase values), the volume of data to work with was very high. Thus, an appropriate data processing method was essential. Subsequently, two different data treatment methodologies were taken into account and compared: Multivariate analysis and artificial neural networks.

#### 2.4.1. Multivariate Analysis

First, a multivariate analysis of the collected data was carried out. To do so, a PCA was preliminarily conducted in order to check whether the obtained data tended to be grouped naturally, showing differences between natural lemons and those that were previously frozen. 

Next, a partial least squares-discriminant analysis (PLS-DA) of the data was performed to discriminate the analyzed samples and identify if there were significant differences among them regarding the obtained variables. The PLS-DA is a regression analysis in which the dependent variable is categorical, which is the class to which the samples belong [46], and the independent variables are the 100 data obtained per analysis (50 modulus values and 50 phase values). The particularity in this methodology is that new independent values are used to test the model. As such, 67% of the data set was used for calibration, and the remaining 33% of the data set was used to test the model [47]. The accuracy of the obtained model was analyzed by the coefficient of determination (R^2^) and the root mean square errors of both cross validation and prediction (RMSCV and RMSEP).

Both PCA and PLS-DA analyses were conducted by using the software SOLO© (Eigenvector Research, Ind., Manson, WA, USA).

#### 2.4.2. ANNs

Alternatively, a study was carried out to detect the possibility of modeling the electrochemical response of the fruits by means of ANNs, as this type of networks is also easy to use, clear, and easily implementable on a PC because it also has low computational requirements. In fact, the potential implementation of ANNs in a microprocessor was of particular interest in this study. Thus, a portable device for in-situ analyses could detect freeze-damage in the fruits when they are still in the fields, thereby meeting all of the low power requirements, easy handling and reliability. Actually, this type of development [48] has already been successfully applied using simplified ANNs, [49,50] which are even simpler, computationally less demanding, quick to program, easy to use and very reliable [51]. The goal was to work with a prediction system that is more flexible, adaptive and versatile than the traditional statistical data treatment methods [52,53]. It was particularly interesting considering that the samples were natural fruits that were subject to a wide set of variables that could have generated more or less diversity among them despite belonging to the same species and the same batch (ripeness, acidity, sugar contents, size, time since they were collected, etc.). 

Type and structure of the ANN were preliminarily fixed by a set of initial trials. Then, a deeper study allowed us to select a specific architecture of the net (layers and neurons in each layer) and also the functions to be applied in each neuron and the algorithms to work within the layers. Data for these studies were appropriately divided by random into three different data sets to be used in the different steps of the ANN design: Training (70%), validation (15%) and test (15%). The first phase (training) allowed us to use the ANN model for the validation and test phases, respectively assessing the obtained model by using both previously used data in the training phase and independent data. Additionally, as overfitting is a probed problem in ANN-based prediction models, a proportional structure of the network was selected, and cross-validation and early-stopping were used when training the model [54]. In this specific case, the accuracy of the obtained ANN was expressed in terms of the correct classification rate (CCR%) and the associated confusion matrix.

ANN modeling was conducted by using the software Alyuda Neurointelligence 2.2 © (Alyuda Research Inc., Cupertino, CA, USA).

## 3. Results

### 3.1. Impedance Spectroscopy Results

**Figure 3:**
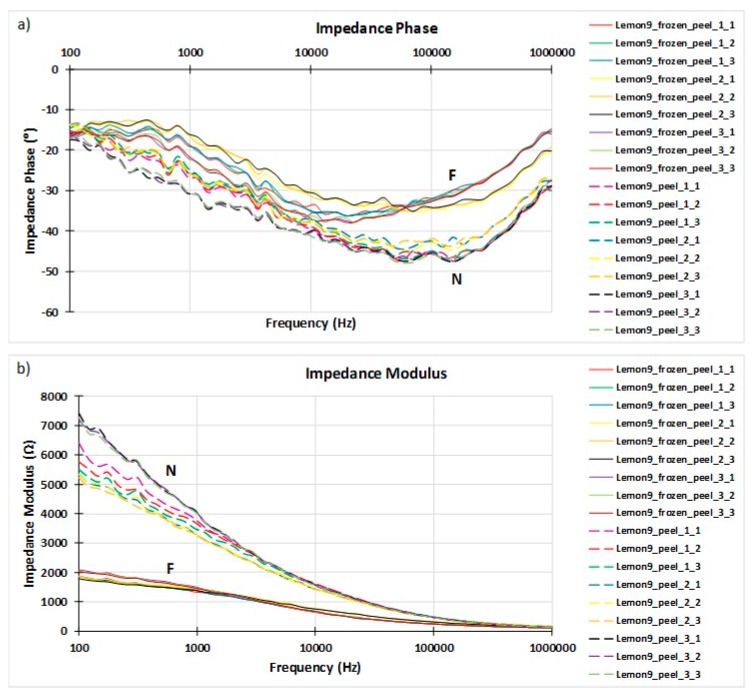
(**a**) Phase and (**b**) modulus of the EIS analyses for lemon number 9 both natural (N) and 12 h after freezing (F) using the sensor directly punctured on the peel.

Once the EIS test was completed, 27,000 data were overall collected (27 tests/sample 10 samples 100 data/test). This data set was appropriately stored in the PC and graphically shown by the software, allowing us to create figures like the ones shown in Figure 3, which represent both the phase and the modulus of the impedance for the selected samples. Graphics showing the Nyquist diagrams were also available in the graphic interface of the software. 

**Figure 4:**
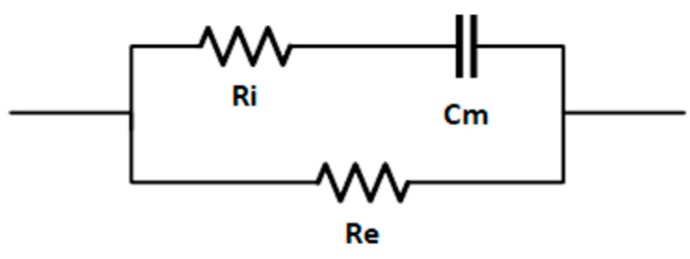
Fricke’s electrical model of biological tissue.

In 1925, Fricke-Morse [55] introduced an equivalent electrical circuit for biological materials (Figure 4) that has been widely used in bioelectrical impedance analysis and other applications such as food technology [22,31,56]. The main advantages of Fricke´s model are its simplicity and direct physical interpretation. In this model, cells are represented by means three elements: Re (resistance of the extracellular fluid), Ri (resistance of the intracellular fluid) and Cm (capacitive component of the cell membrane) [57].

The obtained results respond to this behavior because freezing changes the internal structure of biological materials by rupturing cell membranes and, thus, the impedance of the materials. In Fricke’s model, this impedance is shown in a double way. Firstly, it is shown in the reduction in the capacitive component, as can be seen in the decrease the absolute value of the impedance phase (Figure 3a) due to the breakdown of the cell membrane itself. Secondly, it is shown in the decrease of the impedance modulus (Figure 3b) due to the increase in conductivity in the extracellular fluid generated by the release of intracellular fluid that alters salt concentration after the cells collapsed. 

In general, this behavior was observed in all the analyzed samples; it was most evident in those in which the threshold temperature of freezing was exceeded, and it was dramatically increased in those cases in which the freezing temperature was somewhat more severe.

### 3.2. Multivariate Analysis Results

**Figure 5:**
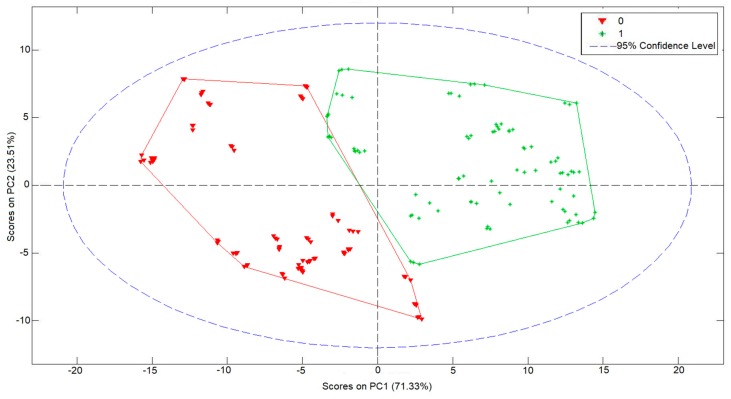
Principal components analysis (PCA) for the studied lemons considering data between two sections. Class 0 (red) represents frozen samples. Class 1 (green) represents the natural ones.

A PCA study of the obtained EIS data allowed us to clearly discriminate samples according to their electrochemical behavior (Figure 5).

This PCA was able to explain up to 71.33% of the variance with just one principal component latent variable and an additional 23.51% if a second one was added. This means that the model was able to explain up to 94.84% with just two principal components latent variables.

**Figure 6:**
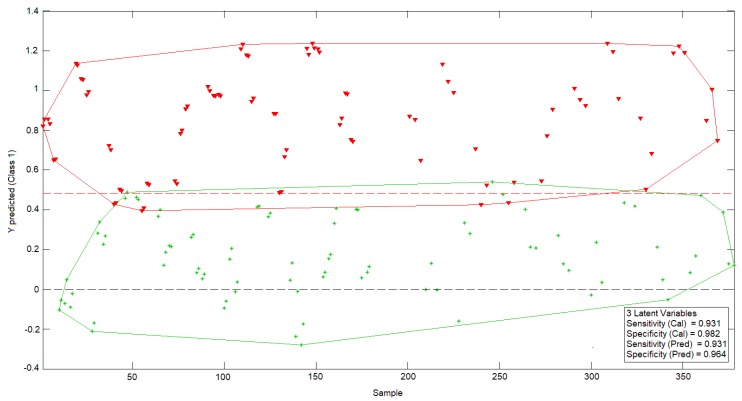
Partial least squares discriminant analysis (PLS-DA) analysis for the same data set. Class 0 (red): Frozen samples. Class 1 (green): Natural samples.

Afterwards, a PLS analysis was carried out in order to obtain a model capable of detecting the freezing phenomena in these samples. Thus, a PLS-DA was specifically used, and the obtained results allowed us to verify that it is possible to classify lemon samples in two different groups (natural and previously frozen), as the model showed high sensitivity for both calibration and prediction phases (Figure 6).

### 3.3. ANNs Results

Finally, an ANN-based model was studied, as this kind of modeling is more flexible, adaptive and does not necessarily respond to a linear behavior pattern. The preliminary results allowed us to observe that it was possible to predict freezing in lemons by ANNs. A more detailed study was able to obtain a 20-13-1 network, this being the best one of the structures suggested by the software. The selected structure was a pyramidal network with three levels or layers: An initial input layer with 20 nodes, a hidden layer with 13 nodes, and an output layer with just one node. This network was run with an on-line back-propagation algorithm and logistic type activation functions in the nodes of both the hidden and the output layers, which proved to be very effective in the discrimination of lemon samples according to whether or not they had experienced a previous frost phenomenon in the last 24 h. The obtained network was able to correctly classify 100% of the samples analyzed (Table 2).

## 4. Conclusions

As the lemon is the most sensitive citrus to cold, establishing strategies to prevent damage and quickly detect the effects of a frost phenomenon is of capital importance. This will help in decision making that aims to minimize wastes and economic losses, as well as to avoid introducing fruits with a lower quality than the expected into the market.

In this study, an EIS technique combined with an adequate data treatment via ANN was analyzed, allowing us to obtain a system able to identify lemon samples that have experienced a freezing phenomenon with a high statistical confidence. Specifically, results showed that this technique was able to successfully classify 100% of the analyzed samples, clearly differentiating samples that had been frozen to those that had not experienced this phenomenon.

This means that this EIS-based technique is a promissory methodology in this specific use and allows us to introduce it as an alternative to the existing laboratory processes that are generally slow, expensive, require complex instruments, and require experienced staff to conduct analyses. 

Additionally, the obtained results suggest that this technique could be also successful for freeze detection in other citrus fruits.

## Figures and Tables

**Table 1:** Specifications of the electrochemical impedance spectroscopy (EIS) measurement system.

Parameter	Specifications
**Frequency range**	1 Hz–1 MHz
**Signal amplitude**	Up to 500 mV
**Type of signal**	Sinusoidal
**Impedance calculation**	Discrete Fourier Transform
**Measured parameters**	Current and Voltage
**Output data**	Modulus and phase of the impedance
**Data set per assay**	Up to 100 data (50 for modulus and 50 for phase)

**Table 2:** Correct classification rates (CCRs) and confusion matrices of the obtained artificial neural network (ANN) model for freeze-damage detection in lemons by EIS.

ANN Architecture: 20-13-1			
**Training**	Validation	Test	Overall
CCR = 100%	CCR = 100%	CCR = 100%	CCR = 100%
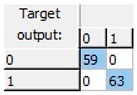	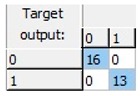	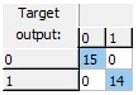	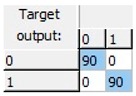

## References

[1] FAO (2017). Citrus Fruit Fresh and Processed Statistical Bulletin 2016.

[2] El-Otmani M., Ait-Oubahou A., Zacarías L., Yahia E.M. (2011). Citrus spp.: Orange, mandarin, tangerine, clementine, grapefruit, pomelo, lemon and lime. Woodhead Publishing Series in Food Science, Technology and Nutrition, Postharvest Biology and Technology of Tropical and Subtropical Fruits.

[3] Zabihi H., Vogeler I., Amin Z.M.Y., Gourabi B.R. (2016). Mapping the sensitivity of citrus crops to freeze stress using a geographical information system in Ramsar, Iran. Weather Clim. Extrem..

[4] Artes F., Artes-Hernandez F., Gómez A.L., Calero F.A., Esnoz A., Nicuesa A.E. (2003). Daños por frío en la postrecolección de frutas y hortalizas. Avances en Ciencias y Técnicas del Frío 1.

[5] Tan E.S., Slaughter D.C., Thompson J.F. (2005). Freeze damage detection in oranges using gas sensors. Postharvest Biol. Technol..

[6] Slaughter D.C., Obenland D.M., Thompson J.F., Arpaia M.L., Margosan D.A. (2008). Non-destructive freeze damage detection in oranges using machine vision and ultraviolet fluorescence. Postharvest Biol. Technol..

[7] Martínez L., Ibacache A., Rojas L. (2008). Daños por heladas en frutales. Tierra Adentro.

[8] Wang C.Y., Gross K.C., Wang C.Y., Saltveir M. (2016). Chilling and freezing injury. The Commercial Storage of Fruits, Vegetables, and Florist and Nursery Stocks. Agriculture Handbook 66.

[9] Snyder R.L., Melo-Abreu J.P., Villar-Mir J.M. (2010). Protección Contra Las Heladas: Fundamentos, Práctica y Economía.

[10] Urbina Vallejo V. (2007). Daños por heladas en frutales. Sintomatología y evaluación. Curs de Valoració de Danys Climatològics i Incendis, Reus-Tarragona, Spain, May 28th–29th 2007 Centre de Formació i Estudis Agrorurals.

[11] Sala J.M., Sanchez-Ballesta M.T., Alférez F., Mulas M., Zacarias L., Lafuente M.T. (2005). A comparative study of the postharvest performance of an ABA deficient mutant of oranges II. Antioxidant enzymatic system and phenylalanine ammonia-lyase in non-chilling and chilling peel disorders of citrus fruit. Postharvest Biol. Technol..

[12] Siboza X.I., Bertling I., Odindo A.O. (2014). Salicylic acid and methyl jasmonate improve chilling tolerance in cold-stored lemon fruit (*Citrus limon*). J. Plant Physiol..

[13] Jha P.K., Xanthakis E., Chevallier S., Jury V., Le-Bail A. (2018). Assessment of freeze damage in fruits and vegetables. Food Res. Int..

[14] Sala J.M., Lafuente M.T. (1999). Catalase in the heat-induced chilling tolerance of cold-stored hybrid Fortune mandarin fruits. J. Agric. Food Chem..

[15] USDA (1999). Arizona California Citrus Loss Adjustment Standards Handbook.

[16] Hatton T.T., Cubbedge R.H. (1978). Separation of frozen grapefruit by using emulsions of differing specific gravities. Proc. Fla. State Hortic. Soc..

[17] Miller W.M., Wardowski W.F., Grierson W., Wardowski W.F., Miller W.M., Hall D.J., Grierson W. (2006). Separation and grading of freeze- damaged fruit. Fresh Citrus Fruits.

[18] Moomkesh S., Ahmad Mireei S., Sadeghi M., Mazeri M. (2017). Early detection of freezing damage in sweet lemons using Vis/SWNIR spectroscopy. Biosyst. Eng..

[19] Obenland D.M., Aung L.H., Bridges D.L., Mackey B.E. (2003). Volatile emissions of navel oranges as predictors of freeze damage. J. Agric. Food Chem..

[20] Gambhir P.N., Choi Y.J., Slaughter D.C., Thompson J.F., McCarthy M.J. (2005). Proton spin–spin relaxation time of peel and flesh of navel orange varieties exposed to freezing temperature. J. Sci. Food Agric..

[21] Fuentes A., Masot R., Fernández-Segovia I., Ruiz-Rico M., Alcañiz M., Barat J.M. (2013). Differentiation between fresh and frozen-thawed sea bream (*Sparus aurata*) using impedance spectroscopy techniques. Innov. Food Sci. Emerg. Technol..

[22] Conesa C., Gracía-Breijo E., Loeff E., Seguí L., Fito P., Laguarda-Miró N. (2015). An Electrochemical Impedance Spectroscopy-Based Technique to Identify and Quantify Fermentable Sugars in Pineapple Waste Valorization for Bioethanol Production. Sensors.

[23] Macdonald J.R., Barsoukov E. (2005). Impedance Spectroscopy. Theory, Experiment and Applications.

[24] Wu L., Ogawa Y., Tagawa A. (2008). Electrical impedance spectroscopy analysis of eggplant pulp and effects of drying and freezing-thawing treatments on its impedance characteristics. J. Food Eng..

[25] Serrano-Pallicer E., Muñoz-Albero M., Pérez-Fuster C., Masot Peris R., Laguarda-Miró N. (2018). Early Detection of Freeze Damage in Navelate Oranges with Electrochemical Impedance Spectroscopy. Sensors.

[26] Grossi M., Riccò B. (2017). Electrical impedance spectroscopy (EIS) for biological analysis and food characterization: A review. J. Sens. Sens. Syst..

[27] Chowdhury A., Bera T.K., Ghoshal D., Chakraborty B. (2017). Electrical Impedance Variations in Banana Ripening: An Analytical Study with Electrical Impedance Spectroscopy. J. Food Process Eng..

[28] Bauchot A.D., Harker F.R., Arnold W.M. (2000). The use of electrical impedance spectroscopy to assess the physiological condition of kiwifruit. Postharvest Biol. Technol..

[29] Figuereido A., Cárdenas N., Rabelo E., Pequeño de Oliveira H. (2017). Determination of mango ripening degree by electrical impedance spectroscopy. Comput. Electron. Agric..

[30] Benavente J., Ramos-Barrado J.R., Heredia A. (1998). A study of the electrical behaviour of isolated tomato cuticular membranes and cutin by impedance spectroscopy measurements. Colloids Surf. A.

[31] Ando Y., Maeda Y., Mizutani K., Wakatsuki N., Hagiwara S., Nabetani H. (2016). Impact of blanching and freeze-thaw pretreatment on drying rate of carrot roots in relation to changes in cell membrane function and cell structure. LWT Food Sci. Technol..

[32] Ando Y., Maeda Y., Mizutani K., Wakatsuki N., Hagiwara S., Nabetani H. (2016). Effect of air-dehydration pretreatment before freezing on the electrical impedance characteristics and texture of carrots. J. Food Eng..

[33] Fuentes A., Vázquez-Gutiérrez J.L., Pérez-Gago M.B., Vonasek E., Nitin N., Barret D.M. (2014). Application of nondestructive impedance spectroscopy to determination of the effect of temperature on potato microstructure and texture. J. Food Eng..

[34] M’hiri N., Veys-Renaux D., Rocca E., Ioannou I., Mihoubi Bourdinova N., Ghoul M. (2016). Corrosion inhibition of carbon steel in acidic medium by orange peel extract and its main antioxidant compounds. Corros. Sci..

[35] Conesa C., Ibáñez J., Seguí L., Fito P., Laguarda-Miro N. (2016). An Electrochemical Impedance Spectroscopy System for Monitoring Pineapple Waste Saccharification. Sensors.

[36] Conesa C., Gil L., Seguí L., Fito P., Laguarda-Miro N. (2017). Ethanol quantification in pineapple waste by an electrochemical impedance spectroscopy-based system and artificial neural networks. Chemom. Intell. Lab. Syst..

[37] Ulrich C., Petersson H., Sundgren H., Björefors F., Krantz-Rülcker C. (2007). Simultaneous estimation of soot and diesel contamination in engine oil using electrochemical impedance spectroscopy. Sens. Actuators B Chem..

[38] Olivati C.A., Riul A., Balogh D.T., Oliveira O.N., Ferreira M. (2009). Detection of phenolic compounds using impedance spectroscopy measurements. Bioprocess Biosyst. Eng..

[39] Martinez Gil P., Laguarda-Miró N., Soto Camino J., Masot Peris R. (2013). Glyphosate detection with ammonium nitrate and humic acids as potential interfering substances by pulsed voltammetry technique. Talanta.

[40] Górski Ł., Sordoń W., Ciepiela F., Kubiak W.W., Jakubowska M. (2016). Voltammetric classifcation of ciders with PLS-DA. Talanta.

[41] Kumar G., Buchheit R.G. (2008). Use of Artificial Neural Network Models to Predict Coated Component Life from Short-Term Electrochemical Impedance Spectroscopy Measurements. Corrosion.

[42] Eddahech A., Briat O., Bertrand N., Delétage J.Y., Vinassa J.M. (2012). Behavior and state-of-health monitoring of Li-ion batteries using impedance spectroscopy and recurrent neural networks. Int. J. Electron. Power Energy Syst..

[43] Conesa C., Seguí L., Laguarda-Miró N., Fito P. (2016). Microwaves as a pretreatment for enhancing enzymatic hydrolysis of pineapple industrial waste for bioethanol production. Food. Bioprod. Process..

[44] Council of Europe (2002). Technical Document. Guidelines on Metals and Alloys Used as Food Contact Materials. Partial Agreement Department in the Social and Public Health Field.

[45] Masot R., Alcañiz M., Fuentes A., Schmidt F.C., Barat J.M., Gil L., Baigts D., Martínez-Máñez R., Soto J. (2010). Design of a low-cost non-destructive system for punctual measurements of salt levels in food products using impedance spectroscopy. Sens. Actuators A Phys..

[46] Wold S., Sjostrom M., Eriksson L. (2001). PLS-regression: A basic tool of chemometrics. Chemom. Intell. Lab..

[47] Legin E., Zadorozhnaya O., Khaydukova M., Kirsanov D., Rybakin V., Zagrebin A., Ignatyeva N., Ashina J., Sarkar S., Mukherjee S. (2019). Rapid Evaluation of Integral Quality and Safety of Surface and Waste Waters by a Multisensor System (Electronic Tongue). Sensors.

[48] Kasuba T. (1993). Simplified fuzzy ARTMAP. AI Expert.

[49] Garcia-Breijo E., Atkinson J., Gil-Sanchez L., Masot R., Ibañez J., Garrigues J., Glanc M., Laguarda-Miro N., Olguin C. (2011). A comparison study of pattern recognition algorithms implemented on a microcontroller for use in an electronic tongue for monitoring drinking waters. Sens. Actuators A Phys..

[50] Rajasekaran S., Vijayalakshmi Pai G.A. (2004). Neural Networks, Fuzzy Logic and Genetic Algorithms: Synthesis and Applications.

[51] Garcia-Breijo E., Garrigues J., Gil Sanchez L., Laguarda-Miró N. (2013). An Embedded Simplified Fuzzzy ARTMAP Implemented on a Microcontroller for Food Classification. Sensors.

[52] Brezmes J., Cabre P., Rojo S., Llobet E., Xilanova X., Correig X. (2005). Discrimination between different samples of olive oil using variable selection techniques and modified fuzzy artmap neural networks. IEEE Sens. J..

[53] Ibáñez Civera J., Garcia Breijo E., Laguarda Miró N., Gil Sánchez L., Garrigues Baixauli J., Romero Gil I., Masot Peris R., Alcañiz Fillol M. (2011). Artificial neural network onto Eight Bit microcontroller for Secchi depth calculation. Sens. Actuators B Chem..

[54] Del Brío B.M., Molina A.S. (2001). Redes Neuronales y Sistemas Borrosos.

[55] Fricke H., Morse S. (1925). The electric resistance and capacity of blood for frequencies between 800 and 4(1/2) million cycles. J. Gen. Physiol..

[56] Damez J.-L., Clerjon S., Abouelkaram S., Lepetit J. (2007). Dielectric behavior of beef meat in the 1–1500 kHz range: Simulation with the Fricke/Cole-Cole model. Meat Sci..

[57] Zhang L., Shen H., Lun Y. (2010). Study on the electric conduction properties of fresh and frozen-thawed grass carp (*Ctenopharyngodon idellus*) and tilapia (*Oreochromis niloticus*). Int. J. Food Sci. Technol..

